# Was the Giant Short-Faced Bear a Hyper-Scavenger? A New Approach to the Dietary Study of Ursids Using Dental Microwear Textures

**DOI:** 10.1371/journal.pone.0077531

**Published:** 2013-10-30

**Authors:** Shelly L. Donohue, Larisa R. G. DeSantis, Blaine W. Schubert, Peter S. Ungar

**Affiliations:** 1 Department of Earth and Environmental Sciences, Vanderbilt University, Nashville, Tennessee, United States of America; 2 Department of Geosciences and Don Sundquist Center of Excellence in Paleontology, Johnson City, Tennessee, United States of America; 3 Department of Anthropology, University of Arkansas, Fayetteville, Arkansas, United States of America; Team ‘Evo-Devo of Vertebrate Dentition’, France

## Abstract

Dramatic environmental changes associated with global cooling since the late Miocene, and the onset of glacial-interglacial cycles in the Pleistocene served as a backdrop to the evolutionary radiation of modern bears (family Ursidae). These environmental changes likely prompted changes in food availability, and triggered dietary adaptations that served as motive forces in ursid evolution. Here, we assess correspondence of dental microwear textures of first and second lower molars with diet in extant ursids. We use the resulting baseline data to evaluate the hypothesis that the Pleistocene giant short-faced bear, *Arctodus simus,* was a bone consumer and hyper-scavenger at Rancho La Brea, California, USA. Significant variation along the tooth row is consistent with functional differentiation, with the second molar serving as a better dietary recorder than the first. Results evince significant variation among species: carnivorous and omnivorous ursids (*Ursus maritimus, U. americanus*) have significantly higher and more variable complexity (*Asfc*) than more herbivorous ones (*Ailuropoda melanoleuca, Tremarctos ornatus, U. malayanus*), and *A. melanoleuca* is differentiated from *U. maritimus* and *U. americanus* by significantly higher and more variable anisotropy (*epLsar*) values. *Arctodus simus* from Rancho La Brea exhibits wear attributes most comparable to its closest living relative (*T. ornatus*), which is inconsistent with hard-object (e.g., bone) consumption, and the hypothesis that short-faced bears were bone consuming hyper-scavengers across their range.

## Introduction

The Pleistocene short-faced bear, *Arctodus simus* was the largest member of the order Carnivora to traverse North America, yet whether this giant was primarily an active predator, opportunistic omnivore, or bone-crushing hyper-scavenger remains unknown. Today, the closest ancestor of *Ar. simus*, the spectacled bear (*Tremarctos ornatus*), is a small, tree-dwelling herbivore/omnivore in South America; and all other modern bears (Family Ursidae) occupy a wide diversity of dietary niches across the globe. Could the dietary adaptability exhibited in past and present ursids have been an important driving force throughout their evolutionary history?

**Figure 1:**
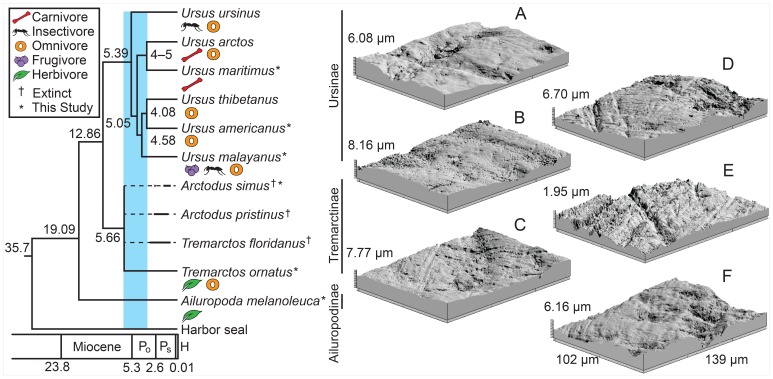
Phylogeny and dietary ecology of Ursidae including three-dimensional microwear photosimulations of analyzed taxa. Phylogeny modified from Krause and colleagues [1] with an update to polar bear origination from Miller and co-authors [2]. The shaded bar highlights the rapid evolutionary radiation of bears, which correlates with climatic and environmental changes. Numbers indicate divergence dates in millions of years. P_o_, Pliocene; P_s_, Pleistocene; H, Holocene. A–F are three-dimensional photosimulations of lower second molars taken at 100× magnification, length and width measurements of photosimulation F correspond to all. A. *Ursus maritimus*, NMNH 512117; B. *Ursus americanus*, UF 28436; C. *Ursus malayanus*, NMNH 151866; D. *Tremarctos ornatus*, NMNH 271418; E. *Ailuropoda melanoleuca*, NMNH 259028; F. *Arctodus simus*, LACMHC 1292.

The Miocene-Pliocene boundary (5.3 Ma) marks an important transition in ursid evolution [1], [2] (Figure 1). Early bears went extinct, and a more modern community, including short-faced species, radiated abruptly following climate induced habitat changes associated with global cooling, increased seasonality [3], and the replacement of forests with open C_4_ grassland habitats – particularly at low latitudes [4]. Given that most modern bears are adaptable opportunists in the face of changing seasons and environments (e.g., [5], [6], [7], [8], [9]), dietary versatility may have allowed ursids to persist during the dramatic habitat fluctuations of the Pleistocene. Clarifying the diets of past ursids, such as *Ar. simus*, is key to understanding the evolutionary history of the family and predicting responses to current and future climate change.

The diet of *Ar. simus* is a topic of debate (e.g., [10], [11]). The giant short-faced bear was initially proposed to be an active predator, running down prey with its long, gracile limbs [12], and capturing/killing victims with large bite forces produced in its short muzzle [13]. Carnivorous tendencies of short-faced bears in Alaska and the Yukon are consistent with elevated δ^15^N values [14], [15], [16], and Fox-Dobbs and colleagues [17] suggest they specialized on caribou. However, others have proposed that the worn teeth and large mandibular muscle attachments of *Ar. simus* are consistent with heavy mastication of plant matter or perhaps crushing of bone [18]. Interpretations of geometric morphometric studies of the mandible further support the idea of large quantities of vegetation in the diet [19]. Yet, other studies of mandibular biomechanics suggest *Ar. simus* was an omnivore [11], [20], [21]. A final hypothesis posits that *Ar. simus* was an obligate scavenger, given fragile and gracile limbs not well-suited to withstanding erratic forces associated with active prey capture; instead, these features allowed it to efficiently travel long distances to search for and acquire large quantities of carrion [22]. Indeed, a large body size [23] would have allowed for an expansive home range, and provided an effective mechanism for carcass defense. With such a discrepancy in dietary interpretations, a new proxy is needed to help clarify the diet of this large ursid.

Dental microwear is a commonly used and effective proxy for dietary reconstruction because it preserves evidence of actual food choice during the last days or weeks of life [24] in contrast to diet during the period of dental development, as with stable isotopes [14], or potential diet, as inferred from functional morphology of cranial and dental elements [25], [26]. Low-magnification microwear studies suggest the potential of microwear for retrodicting bear diets [27], [28], but these studies did not include either omnivorous brown bears (*Ursus arctos*) or omnivorous black bears (*U. americanus*, *U. thibetanus*).

There are other studies of ursid microwear. *Ursus arctos* and extinct European cave bears (*U. spelaeus*) have been studied at 200× by scanning electron microscopy to infer both dietary differences and functionality of the first molar [29]. But as with other microwear studies on bears, the focus was exclusively on carnassials (e.g., lower first molars) and upper first molars, even though more posterior teeth likely used in crushing/grinding might capture other dietary information especially relevant for omnivorous ursids.

A relatively new approach to dental microwear, texture analysis, has been especially valuable for characterizing subtle differences in dietary patterns within and between species. This combines scanning white light confocal microscopy with scale sensitive fractal analysis to quantify surface textures using an automated and repeatable method that reduces observer bias [30], [31], [32]. It has been used to successfully differentiate discrete dietary niches within primates (e.g., [32], [33]), bovids [34], marsupials [35], xenarthrans [36], and carnivores [37], [38], and may be able to differentiate diets within populations, including dietary differences across seasons and between sexes (e.g., [34], [39]).

Here, we report the first application of dental microwear texture analysis (DMTA) to ursids. We analyze five extant species to construct a modern baseline: the specialist bamboo-consuming giant panda (*Ailuropoda melanoleuca*) [8], [40], [41], herbivorous/omnivorous spectacled bear (*Tremarctos ornatus*) [5], frugivorous/insectivorous/omnivorous sun bear (*Ursus malayanus*) [7], [42], omnivorous American black bear (*U. americanus*) [6], [43], [44], and carnivorous polar bear (*U. maritimus*) [9], [45], [46], [47] (see File S1 for in-depth dietary descriptions, and summary Table S1). We develop this modern baseline to address the following questions: (i) do modern bears with disparate diets have distinct dental microwear textures, and (ii) does dental microwear vary significantly between lower first and second molars given functional differentiation of teeth? We also examine *Ar. simus* microwear and compare results to our baseline to evaluate the hypothesis that the Pleistocene short-faced bear at Rancho La Brea, California (USA) was a bone-crushing hyper-scavenger.

## Materials and Methods

Modern ursid specimens chosen for analysis are adult, wild-caught, and include detailed provenience metadata. Further, omnivorous *U. americanus* and carnivorous *U. maritimus* have large geographic ranges; thus, specimens from multiple locations within each range were chosen in order to capture maximum dietary variation. Specimens were sampled from publicly accessible collections in the United States, including the following: the American Museum of Natural History (AMNH) in New York, NY, the Florida Museum of Natural History (FLMNH) in Gainesville, FL, and the Smithsonian Institute National Museum of Natural History (NMNH) in Washington DC. Extinct *Ar. simus* specimens were sampled from the publicly accessible collections at the Los Angeles County Museum of Natural History, Page Museum, Hancock Collection (LACMHC) in Los Angeles, CA, and represent a time span of 26,427 (Pit 9) –35,370 (Pit 77) radiocarbon years before present [48]; however, several specimens lacking pit information were also sampled. Based on the total age estimates of the site, all specimens lived during the end of the Wisconsin glaciation, up to 50,000 radiocarbon years before present [48]. All research was done in accordance with the ethics guidelines set by the Society of Vertebrate Paleontology, including the study of modern and fossil specimens from public museum collections that were sampled with the permission of museum curators and/or collections managers at each above mentioned institution. All sampled specimens are listed in the (Table S10), along with complete information for each repository.

Molding and casting procedures followed conventional microwear methods [32], [33], [38], and involved thorough cleaning of tooth facets with acetone soaked cotton swabs prior to application of regular body polyvinylsiloxane dental impression material (President’s Jet, Coltene-Whaledent Corp., Altstätten, Switzerland). At Vanderbilt University, molds were reinforced with polyvinylsiloxane dental putty (President’s Putty, Coltene-Whaledent Corp., Altstätten, Switzerland) and replicas cast using high-resolution epoxy (Epotek 301, Epoxy Technologies Corp., Billerica, MA, USA).

**Figure 2:**
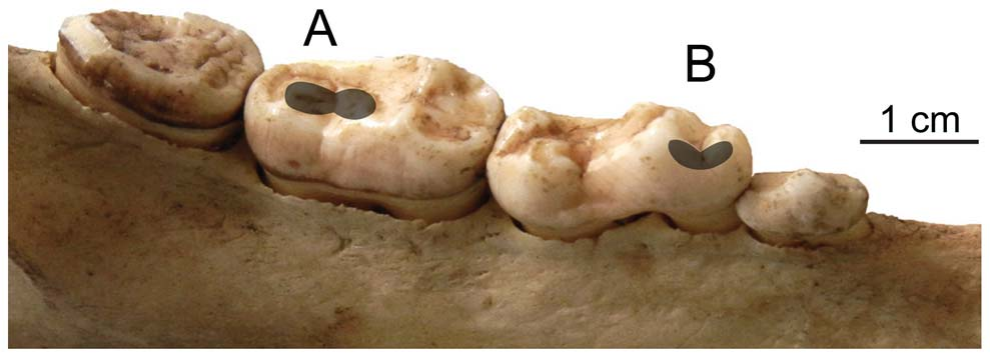
Depiction of ursid tooth facets analyzed for dental microwear. Pictured: *Ursus americanus* (NMNH 198391), left mandible, buccal view, anterior is to the right. Shading indicates scanned regions. A. Second molar (m2) hypoconulid, mesial facet. B. First molar (m1) protoconid, buccal facet.

The buccal facet of the lower first molar (m1) protoconid was analyzed to evaluate shearing functionality, homologous to other carnivorans [37], [38]; the mesial facet of the lower second molar (m2) hypoconulid was analyzed to assess crushing/grinding functionality, analogous to Phase II facets in primates [33] (Figure 2). Tooth replicas were scanned with a Sensofar Plu white-light scanning confocal profiler (Solarius Development, Inc., Sunnyvale, CA) using a 100× objective lens at the University of Arkansas. One set of four adjacent scans per facet was sampled, for a total scanned area of 204×276 µm. Scanned surfaces were leveled using Solarmap universal software (Solarius Development, Sunnyvale, CA, USA) and small artifacts (e.g., dust) removed via point deletion editing and so excluded from surface texture analyses.

Several dental microwear attributes were analyzed to infer diet. Complexity (*Asfc*) is a measure of surface roughness across scales of observation [32], [33]. Heavy pitting due to hard nut or bone consumption should produce a high *Asfc* value. Anisotropy (*epLsar*) quantifies the orientation of microwear surface textures [32], [33]. High anisotropy indicates constrained tooth-tooth movements, as expected in repeated shearing of tough foods, such as meat or leaves. Textural fill volume (*Tfv*) provides an indication of microwear feature sizes. It is calculated as the difference between summed volumes of square cuboids (10 µm and 2 µm on a side, respectively), that “fill” a surface [30]. Individuals with many deep features in the 2–10 µm size range tend to have high *Tfv* values. Scale of maximum complexity (*Smc*) is a measure of the scale at which roughness increase tails off; and high values are typically found on surfaces that lack fine-scale, or small features. Heterogeneity of complexity (*HAsfc*) reflects variation in complexity across the sampled surface. It is calculated as variance in complexity of subdivisions of the sampled area (in grids of 3×3 and 9×9) as compared with the complexity of the whole surface [30]. High *HAsfc* values may suggest variation in material properties of consumed food particles (e.g., size, hardness). Scott and colleagues [30] describe each microwear texture attribute in detail.

All data analyses were completed using ToothFrax and SFrax (Surfract Corp., http://www.surfract.com/) software. Median values were calculated for the four scans of each specimen and used to represent each tooth analyzed [30]. DMTA attributes are commonly not normally distributed (Shapiro Wilk tests, Table S2); thus, nonparametric tests were employed. To assess differential microwear patterning across the tooth row, lower first and second molars from like individuals were compared using a pairwise Wilcoxon signed-rank test (non-parametric), and paired Student’s t-test (parametric) where applicable. Character means among species were compared using Kruskal-Wallis tests following Dunn’s procedure [49]. In order to account for the possibility of Type I and II errors, multiple analyses of variance (MANOVA) were performed on ranked data [50], followed by ANOVA’s and *post-hoc* Fisher’s (LSD) and Tukey’s (HSD) tests on individual DMTA attributes to assess sources of variance, similar to DeSantis and colleagues [38]. Differences in variances between taxa were also assessed. Raw data were median-transformed for Levene’s test following the equation X′ = |X – median(X)/median(X)|, as described by Plavcan and Cope [51]. A MANOVA, ANOVAs and multiple comparisons tests were then run on transformed data.

## Results

**Figure 3:**
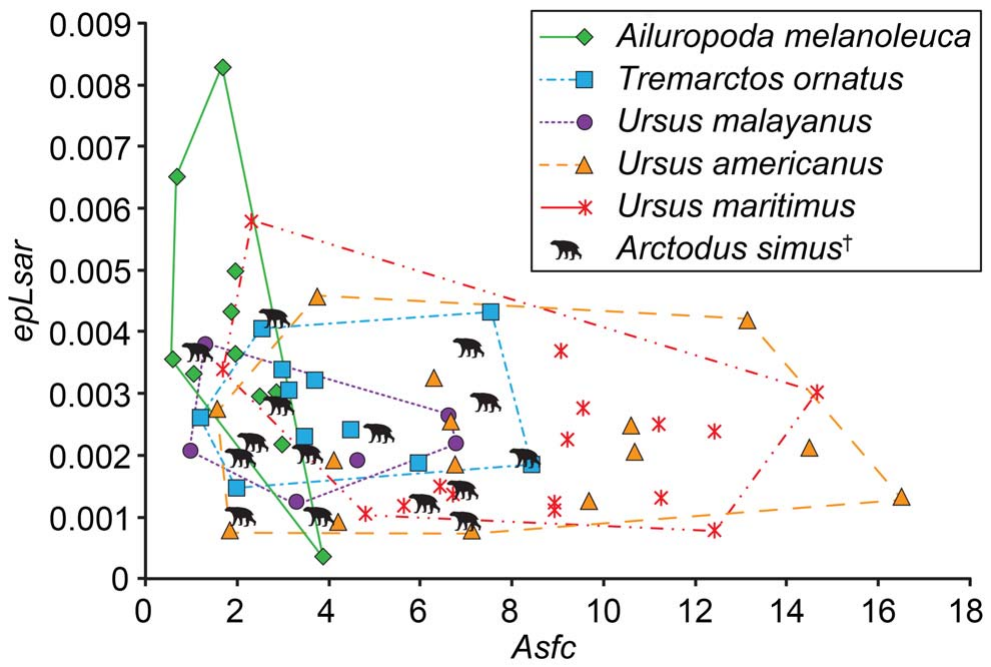
Bivariate plot of complexity (*Asfc*) vs. anisotropy (*epLsar*) for lower second molars of ursids. Polygons enclose data points.

Results are presented in Tables 1, 2, 3, Tables S2, S3, S4, S5, S6, S7, S8, S9, S10, Figures 1 and 3, and Figure S1 and S2. There are significant differences between lower first and second molars of individuals (Table 1). DMTA attributes correlated with diet exhibit more differences between disparate dietary niches in ursid m2s; thus, m1 results are reported only in Figure S1 and Tables S3, S4, S5. Significant Kruskal-Wallis and MANOVA (*P*<0.05) results indicate differences in DMTA attributes among species. Dunn’s procedure indicates that bamboo-consuming *A. melanoleuca* differs from carnivorous *U. maritimus* by significantly lower *Tfv* values and from both *U. maritimus* and omnivorous *U. americanus* by significantly higher *epLsar* values and lower *Asfc* values (Table 2). Complexity values further differentiate species with differing diets, as herbivorous/omnivorous *T. ornatus* exhibits significantly lower values than both *U. maritimus* and *U. americanus,* and frugivorous/insectivorous/omnivorous *U. malayanus* exhibits significantly lower values than *U. maritimus* (Table 2). *Arctodus simus* exhibits DMTA attributes with means most similar to herbivorous/omnivorous *T. ornatus* and omnivorous *U. americanus* (Table S7), and significant differences (*P*<0.05) in at least one DMTA attribute distinguish it from all other taxa. Specifically, *Ar. simus* differs from carnivorous *U. maritimus* with lower *Asfc* and higher *Tfv* values, from bamboo-consuming *A. melanoleuca* with higher *Asfc* and *Tfv* and lower *epLsar* values and from frugivorous/insectivorous/omnivorous *U. malayanus* with higher *Tfv* values (*P*<0.05; Table S7, Figure S2). Results from Tukey’s (HSD) *post-hoc* tests of ranked data are consistent with the results from Dunn’s procedure, with two exceptions (Table S9). Fisher’s (LSD) tests further suggest variation in *Asfc* values between herbivorous/omnivorous *T. ornatus* and bamboo-consuming *A. melanoleuca*, and omnivorous *U. americanus* and frugivorous/insectivorous/omnivorous *U. malayanus.* We consider these results suggestive or of marginal significance because Fisher’s (LSD) test is less conservative than Tukey’s (HSD) test.

**Table 1:** Descriptive statistics of dental microwear attributes used to differentiate diet in bears.

			*Asfc*		*epLsar*		*Tfv*	
Species	Tooth	*n*	mean	SD	mean	SD	mean	SD
*Ailuropoda melanoleuca*	m1	15	5.705	5.741	0.0040	0.0051	3520	5432
	m2	11	1.996	1.010	0.0039	0.0021	8230	5507
*Tremarctos ornatus*	m1	15	4.348	3.245	0.0013	0.0035	**8543** *	5248
	m2	11	4.172	2.288	0.0028	0.0009	**12987** *	4410
*Ursus malayanus*	m1	7	**1.44** *	0.719	**0.0030** *	0.0012	11057	3309
	m2	6	**3.96** *	2.519	**0.0023** *	0.0009	10639	3715
*Ursus americanus*	m1	16	**4.21** *	3.035	**0.0006** *	0.0025	**9992** *	4232
	m2	15	**7.85** *	4.576	**0.0022** *	0.0011	**12192** *	4528
*Ursus maritimus*	m1	15	**4.91** *	2.444	0.0020	0.0041	**10650** *	3812
	m2	16	**8.49** *	3.640	0.0022	0.001	**13036** *	2136
*Arctodus simus* †	m1	15	3.350	1.962	0.0025	0.0008	**13382** *	2513
	m2	16	4.586	2.295	0.0022	0.0010	**15028** *	1753

* Indicates significant differences (*P*<0.05, Wilcoxon signed-rank test or paired Student’s t-test, where applicable) between m1 and m2 values;

† denotes the extinct taxon; SD, standard deviation, *n*, number of individuals sampled; *Asfc*, area-scale fractal complexity; *epLsar*, anisotropy; *Tfv*, textural fill volume.

**Table 2:** Pairwise differences for microwear attributes of extant ursid lower second molars (Dunn’s procedure).

	*T. ornatus*	*U. malayanus*	*U. americanus*	*U. maritimus*
***Asfc***				
*A. melanoleuca*	−13.00	−11.20	−**26.36** *	−**29.86** *
*T. ornatus*		1.80	−**13.36** *	−**16.86** *
*U. malayanus*			−15.17	−**18.67** *
*U. americanus*				−3.5
***EpLsar***				
*A. melanoleuca*	8.91	15.98	**17.75** *	**18.51** *
*T. ornatus*		7.08	8.84	9.60
*U. malayanus*			1.77	2.52
*U. americanus*				0.75
***Tfv***				
*A. melanoleuca*	−14.09	−3.58	−12.84	−**13.47** *
*T. ornatus*		10.52	1.25	0.62
*U. malayanus*			−9.27	−9.90
*U. americanus*				−0.63

* Significant values are noted in bold text (*P*<0.05) and represent analyses performed absent of the Bonferroni correction; *Asfc*, area-scale fractal complexity; *epLsar*, anisotropy; *Tfv*, textural fill volume.

**Table 3:** Pairwise differences of variance between lower second molars of all extant and extinct bears.

	*T. ornatus*	*U. malayanus*	*U. americanus*	*U. maritimus*	*Ar. simus* †
***Asfc***					
*A. melanoleuca*	0.134	0.090	−**0.405** *	−**0.476** *	−0.082
*T. ornatus*		−0.044	−**0.538** *	−**0.610** **	0.051
*U. malayanus*			−**0.495** *	−**0.566** *	−0.008
*U. americanus*				−0.072	−**0.487** *
*U. maritimus*					−**0.558** **
***epLsar***					
*A. melanoleuca*	**0.522** **	**0.578** *	**0.466** **	**0.411** *	**0.510** **
*T. ornatus*		0.056	−0.056	−0.111	−0.012
*U. malayanus*			−0.112	−0.167	−0.067
*U. americanus*				−0.055	0.044
*U. maritimus*					0.100

* Indicates significant (*P*<0.05) Fisher’s (LSD) tests;

** indicates significant (*P*<0.05) Fisher’s (LSD) and Tukey's (HSD) tests; *Asfc*, area-scale fractal complexity; *epLsar*, anisotropy.

† Denotes the extinct taxon. Data transformed for Levene’s test, X′ = |X – median(X)/median(X)|following Plavcan and Cope [51].

Assessment of variability among character distributions in modern ursids suggested significant (*P*<0.05) variation for a MANOVA of Levene-transformed *Asfc, epLsar,* and *Tfv* data. Individual ANOVAs on *Asfc* and *epLsar* showed significant variation (*P*<0.05). Anisotropy of bamboo-consuming *A. melanoleuca* is more variable than herbivorous/omnivorous *T. ornatus,* omnivorous *U. americanus* and extinct *Ar. simus* based on Tukey’s (HSD) test (*P*<0.05), and marginally so compared with frugivorous/insectivorous/omnivorous *U. malayanus* and carnivorous *U. maritimus* based on Fisher’s (LSD) tests (Table 3). Complexity of *U. americanus* is marginally more variable than all extant bears (except *U. maritimus*). Similarly, *Asfc* of *U. maritimus* is significantly more complex than *T. ornatus,* and marginally more complex than *A. melanoleuca* and *U. malayanus. Arctodus simus* has significantly and marginally less variable *Asfc* than carnivorous *U. maritimus* and omnivorous *U. americanus*, respectively, suggesting that it is overall most similar to herbivorous/omnivorous *T. ornatus* (Table 3). Raw data for all ursid specimens analyzed in this study are reported in the (Table S10).

## Discussion

### Dental Microwear of Extant Ursids

Dental microwear differences among m2s of the various ursid taxa correlate well with observed differences in diets of extant bears. This strongly suggests the potential of texture analysis to retrodict diet in extinct bear species (Figure 3). *Ailuropoda melanoleuca* has low *Asfc* values, consistent with oral processing of bamboo leaves, shoots, and peeled stems. Prior to consumption of bamboo stems, the stalk is held by the paw and the hard exterior peeled off using the anterior molars and teeth. The tough peeled stalk is bitten into bite-sized pieces and chewed with the more posterior teeth [8]. High *epLsar* values reflect the high silica content of bamboo [52], and are consistent with previous SEM [53] and optical steromicroscopic [27], [28] microwear studies finding high numbers of scratches and infrequent pitting in *A. melanoleuca*. Our results are also in-line with analyses of dental morphology, as the derived, cuspidate cheek teeth of pandas suggest consumption of foods requiring substantive mechanical processing [26]. Consumption of large quantities of plant matter is also evidently reflected in moderate *Asfc* values in *T. ornatus* and *U. malayanus,* who process tough leafy matter, seeded or pitted fruits, and some insects [5], [7], [42]. In contrast to more herbivorous bears, the high variance of *Asfc* values exhibited by *U. americanus* (Table 3) may reflect the high degree of dietary adaptability by this species across geographic regions (Alaska vs. Florida, Table S10) and seasons. Individuals displaying high *Asfc* values likely consumed brittle or hard food (e.g., nuts or berries with hard seeds [6], [43], [44]) prior to death. *Ursus maritimus* also has highly variable *Asfc* values, similar to *U. americanus,* despite a diet consisting primarily of soft seal flesh and blubber [9]. High *Asfc* values are likely the result of bone consumption during scavenging [9], [45] or consumption of terrestrial food sources including coastal and freshwater fish, and berries during summer months [46], [47].

Inferring diet from dental microwear textures of lower first molars of modern bears is complicated by the use of the carnassial in food acquisition. Ursids often use their forelimbs to stabilize food items while grabbing, tearing, or cracking food with their carnassials [5], [7], [8], [40]. Thus, it is likely that microwear attributes on the m1 are a reflection of both differences in food acquisition and processing, and it is difficult to parse the two. This may explain the lack of distinguishable *Asfc* values for lower carnassials (i.e., m1s) between all modern bears analyzed (excluding *U. malayanus*), despite differences in physical properties of consumed food between species. *Ailuropoda melanoleuca*, in particular, uses its anterior teeth (including the m1) during bamboo processing prior to consumption [8], [40]. Higher *Asfc* scores of the m1 likely reflect peeling and biting of bamboo stalks, while the m2 is used primarily for chewing. In contrast, *U. malayanus* likely avoids using the m1 during food acquisition, as low *Asfc* values do not reflect the high proportion of fruit and insects in its diet [7], [42]. Alternatively, low *Asfc* values in *U. malayanus* may indicate preferential use of the m1 on softer/tougher food items, perhaps with high silica content [52], or they may not be representative of the species given their small sample size (*n* = 6). Overall, dental microwear attributes of the carnassial shearing facet (m1) do not relate to known dietary differences of modern bears, and thus the m2 is likely a better proxy for diet in extinct ursids.

### Dental Microwear of *Arctodus simus*

Dental microwear texture analysis of lower second molars suggests that *Ar. simus* was not a durophagous hyper-scavenger of carcasses at Rancho La Brea, CA. Hyper-scavengers are expected to crunch bone at least some of the time, and thus exhibit high *Asfc* values, similar to modern hyenas (*Crocuta crocuta,* mean *Asfc = *9.315, [38]). Significantly lower (*P*<0.05, Wilcoxon signed rank test) values in both the m1 and the m2 of *Ar. simus* in comparison to *C. crocuta* do not support durophagy in this ursid at La Brea. Further, *Ar. simus* has significantly lower *Asfc* values (*P*<0.05, Tukey’s (HSD) test) than extant *U. maritimus,* which participates in some carcass scavenging of marine mammals on sea ice [9], [45]. Although microwear differentiates between the physical properties of consumed food and suggests that *Ar. simus* was not a hard-object feeder at La Brea, it cannot identify the trophic level of the short-faced bear. Stable carbon and nitrogen isotopes suggest *Ar. simus* from northwestern North America was a carnivore [14], [15], [16], but isotopic values have not been reported for mid-lower North America, and the diet of *Ar. simus* likely varied by region [10], similar to modern ursids (e.g., [5], [6], [7], [8], [9]).

*Arctodus simus* likely included some plant and animal material in its diet, and at La Brea it avoided hard/brittle food items. Morphologically, *Ar. simus* exhibits characters common to herbivorous bears, including cheek teeth with large surface areas, a deep mandible, and large mandibular muscle attachments [11], [21]. Because herbivorous carnivorans lack an efficient digestive tract for breaking down plant matter via microbial action [54], they must break down plant matter via extensive chewing or grinding, and thus possess features to create a high mechanical advantage of the jaw [21], [25]. While the presence of features indicating herbivory in *Ar. simus* likely relate to diet, the close phylogenetic relationship to the herbivorous/omnivorous spectacled bear must also be considered, as these traits may be an ancestral condition of the group. Regardless, gross tooth wear suggests consumption of at least some plant matter in the diet of *Ar. simus* [18], [20]. Despite presumed inclusion of both plant and animal material in the diet of *Ar. simus*, individuals from La Brea were likely less generalized than modern *U. americanus*, based on relatively constrained *Asfc* values that remain consistent throughout the late Pleistocene.

### Extinction Implications

Previous research concerning the extinction of numerous large-bodied carnivorans at Rancho La Brea examined incidence of tooth breakage, and suggested that times were ‘tough’ as food shortages led carnivorans to more fully utilize carcasses, including bone consumption [55]. However, prior texture analysis from DeSantis and colleagues [38], found a lack of evidence for high levels of carcass utilization in the American lion (*Panthera atrox*) and saber-toothed cat (*Smilodon fatalis*). Similarly, Schmitt [56] found no evidence for ‘tough times’ in the dire wolves (*Canis dirus*), which exhibited wear less complex than the durophagous, scavenging African wild dog (*Lycaon pictus*). Here, there is no support for durophagous carcass utilization by a single *Arctodus simus* individual. While it is possible that *Ar. simus* engaged in scavenging of flesh from recent kills, it is expected that a scavenger would consume at least some bone. DeSantis and colleagues [38] suggest that high incidences of broken teeth in extinct carnivores from La Brea were potentially inflicted during the acquisition of larger prey abundant on Pleistocene landscapes, rather than through bone consumption. Regardless of the cause of broken teeth in extinct carnivorans, the microwear texture data for cats [38], dogs [56], and bears collectively offer no evidence that food shortages are to blame for the demise of large bodied carnivorans at Rancho La Brea. Collectively, these ursid data suggest that the giant short-faced bear was likely not a bone-crushing hyper-scavenger throughout its range.

## Supporting Information

Figure S1 **Bivariate plot of complexity (** ***Asfc*** **) vs. anisotropy (** ***epLsar*** **) for lower first molars of ursids.** Polygons enclose data points.
(PDF)

Figure S2 **Raw data for** ***Asfc, epLsar,*** **and** ***Tfv*** **for lower second molars of ursids.** Black data points indicate mean value for each species.
(PDF)

Table S1 **Review of diet in modern ursids, with predicted microwear based on physical properties of known diet.**
(PDF)

Table S2 ***P*** **-values for Shapiro-Wilk tests for normality for lower first (m1) and second (m2) molars.**
(PDF)

Table S3 **Descriptive statistics for dental microwear attributes that did not exhibit significant differences among lower first (m1) and second (m2) molars.**
(PDF)

Table S4 **Table of pairwise differences of Dunn’s procedure for dental microwear attributes of lower first molars of extant ursids.**
(PDF)

Table S5 **Table of pairwise differences (Dunn’s procedure) for lower first molar dental microwear attributes of extant ursids and** ***Arctodus simus.***
(PDF)

Table S6 **Table of pairwise differences (Dunn’s procedure) for extant ursid lower second molar dental microwear attributes, exhibiting no significant differences.**
(PDF)

Table S7 **Table of pairwise differences (Dunn’s procedure) for lower second molars of extant ursids and** ***Arctodus simus.***
(PDF)

Table S8 **Table of pairwise difference for** ***post-hoc*** **tests on significant (** ***P*** **<0.05) ANOVAs of extant ursid lower first molars.**
(PDF)

Table S9 **Table of pairwise difference for** ***post-hoc*** **tests on significant (** ***P*** **<0.05) ANOVAs of extant ursid lower second molars.**
(PDF)

Table S10 **Data for lower first (m1) and second (m2) lower molars of analyzed specimens.**
(PDF)

File S1 **Supplemental dietary background of extant Ursids.**
(PDF)
