# Supplementary material for: Was the Giant Short-Faced Bear a Hyper-Scavenger? A New Approach to the Dietary Study of Ursids Using Dental Microwear Textures
Source: PLoS One. 2013 Oct 30;8(10):e77531. doi: 10.1371/journal.pone.0077531 (PMC3813673; doi:10.1371/journal.pone.0077531)

Figure S1. Bivariate plot of complexity (*Asfc*) vs. anisotropy (*epLsar*) for lower first molars of ursids. Polygons enclose data points.

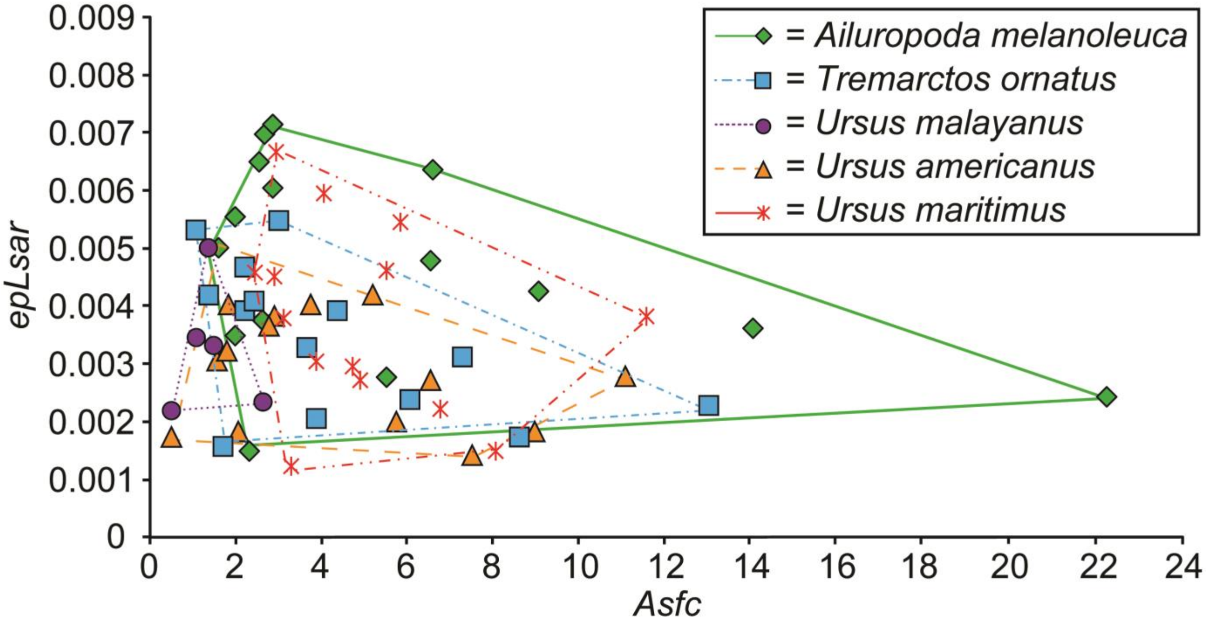

Supplement: Figure S1 — Bivariate plot of complexity ( Asfc ) vs. anisotropy ( epLsar ) for lower first molars of ursids. Polygons enclose data points. (PDF) [file pone.0077531.s001.pdf]
