# Supplementary material for: Was the Giant Short-Faced Bear a Hyper-Scavenger? A New Approach to the Dietary Study of Ursids Using Dental Microwear Textures
Source: PLoS One. 2013 Oct 30;8(10):e77531. doi: 10.1371/journal.pone.0077531 (PMC3813673; doi:10.1371/journal.pone.0077531)

Figure S2. Raw data for *Asfc, epLsar,* and *Tfv* for lower second molars of ursids. Black data points indicate mean value for each species.

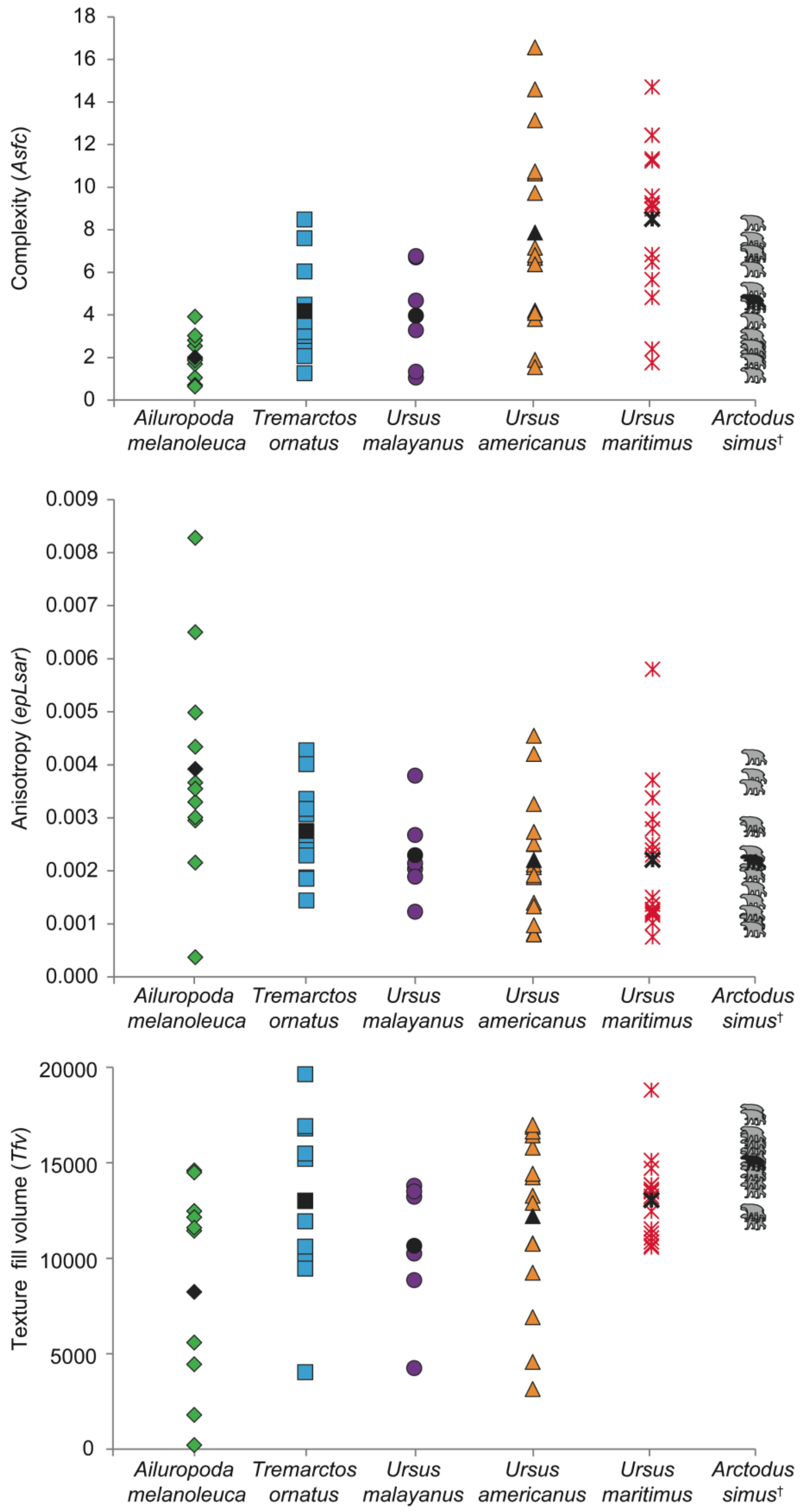

Supplement: Figure S2 — Raw data for Asfc, epLsar, and Tfv for lower second molars of ursids. Black data points indicate mean value for each species. (PDF) [file pone.0077531.s002.pdf]
