# Supplementary material for: Was the Giant Short-Faced Bear a Hyper-Scavenger? A New Approach to the Dietary Study of Ursids Using Dental Microwear Textures
Source: PLoS One. 2013 Oct 30;8(10):e77531. doi: 10.1371/journal.pone.0077531 (PMC3813673; doi:10.1371/journal.pone.0077531)
Supplement: Table S1 — Review of diet in modern ursids, with predicted microwear based on physical properties of known diet. (PDF) [file pone.0077531.s003.pdf]

Table S1. Review of diet in modern ursids, with predicted microwear based on physical properties of known diet.

Species	Diet Category	Main dietary components	Prediction of Microwear		Source
			Complexity (*Asfc*)	Anisotropy (*epLsar*)	
Ursus maritimus	Carnivore	Ringed seals, bearded seals, walruses, carrion, fish, berries	Low when soft seal blubber and flesh consumed; infrequent high values from terrestrial berries and fish consumption	Low to moderate with consumption of soft seal blubber and flesh	[1], [2], [3], [4]
Ursus americanus	Omnivore	Berries, nuts, insects, herbaceous plants, small vertebrates	High in fall with consumption of nuts, low in spring with consumption of herbaceous plants	High in spring with consumption of herbaceous plants, low other times of year	[5], [6], [7]
Ursus malayanus	Insectivore – Frugivore – Omnivore	Termites, beetles, larvae, tree fruits, honeycomb,	Low when tree fruits consumed; moderate-high when insects consumed	Low due to lack of foods with high silica content, insects not thoroughly chewed	[8], [9]
Tremarctos ornatus	Herbivore – Omnivore	Bromeliad hearts, palm fronds, tree wood, tree fruits: e.g. fig, caper	Low due to avoidance of hard/brittle plant components	Moderate to high during consumption of tough bromeliad hearts and palm fronds	[10]
Ailuropoda melanoleuca	Specialist Herbivore	Bamboo leaves, pith, shoots	Low due to avoidance of hard/brittle bamboo components	High due to high silica content of bamboo, bamboo chewed thoroughly	[11], [12], [13]
