# Supplementary material for: Was the Giant Short-Faced Bear a Hyper-Scavenger? A New Approach to the Dietary Study of Ursids Using Dental Microwear Textures
Source: PLoS One. 2013 Oct 30;8(10):e77531. doi: 10.1371/journal.pone.0077531 (PMC3813673; doi:10.1371/journal.pone.0077531)
Supplement: Table S2 — P -values for Shapiro-Wilk tests for normality for lower first (m1) and second (m2) molars. (PDF) [file pone.0077531.s004.pdf]

Table S2. ***P*-values for Shapiro-Wilk tests for normality for lower first (m1) and second (m2) molars.**

Species	Tooth	*Asfc*	*epLsar*	*Smc*	*Tfv*	*Hasfc*$_{(3x3)}$	*Hasfc*$_{(9x9)}$
Ailuropoda melanoleuca	m1	**0.0003***	**< 0.0001***	**0.003***	**0.0002***	0.562	0.287
	m2	0.776	0.705	**0.001***	0.055	0.800	0.083
Tremarctos ornatus	m1	**0.011***	**< 0.0001***	**0.001***	0.157	0.460	**0.031***
	m2	0.257	0.796	**0.002***	0.841	**0.034***	0.570
Ursus malayanus	m1	0.579	0.702	0.113	0.641	0.877	0.347
	m2	0.299	0.606	**0.008***	0.191	**0.024***	0.882
Ursus americanus	m1	0.059	**< 0.0001***	**0.009***	0.653	0.276	**0.001***
	m2	0.542	0.252	**< 0.0001***	0.082	0.628	0.123
Ursus maritimus	m1	**0.013***	**< 0.0001***	**< 0.0001***	0.067	**0.015***	**0.028***
	m2	0.761	**0.023***	**< 0.0001***	**0.046***	**0.028***	**0.013***
Arctodus simus†	m1	0.148	0.179	**< 0.0001***	0.352	0.233	**0.036***
	m2	0.218	0.216	**< 0.0001***	0.403	0.676	0.657

*Significant values are noted in bold text (p<0.05); † denotes the extinct taxon; *Asfc*, area-scale fractal complexity; *epLsar*, anisotropy; *Smc*, scale of maximum complexity; *Tfv*, textural fill volume; *HAsfc*$_{(3x3)}$, *HAsfc*$_{(9x9)}$ heterogeneity of complexity in a 3x3 and 9x9 grid, respectively
