# Supplementary material for: Was the Giant Short-Faced Bear a Hyper-Scavenger? A New Approach to the Dietary Study of Ursids Using Dental Microwear Textures
Source: PLoS One. 2013 Oct 30;8(10):e77531. doi: 10.1371/journal.pone.0077531 (PMC3813673; doi:10.1371/journal.pone.0077531)
Supplement: Table S3 — Descriptive statistics for dental microwear attributes that did not exhibit significant differences among lower first (m1) and second (m2) molars. (PDF) [file pone.0077531.s005.pdf]

Table S3. Descriptive statistics for dental microwear attributes that did not exhibit significant differences among lower first (m1) and second (m2) molars.

			Smc		***Hasfc(3x3)***		***Hasfc(9x9)***	
**Species**	**Tooth**	***n***	mean	SD	mean	SD	mean	SD
Ailuropoda melanoleuca	m1	15	0.248	0.107	0.387	0.105	0.607	0.170
	m2	11	0.203	0.081	0.436	0.116	0.798	0.215
Tremarctos ornatus	m1	15	0.211	0.080	0.479	0.116	0.810	0.170
	m2	11	0.226	0.105	0.490	0.148	0.754	0.202
Ursus malayanus	m1	7	0.273	0.142	0.403	0.046	0.628	0.104
	m2	6	0.202	0.083	0.414	0.167	0.795	0.243
Ursus americanus	m1	16	0.239	0.097	0.552	0.130	0.875	0.875
	m2	15	0.172	0.052	0.536	0.151	0.870	0.363
Ursus maritimus	m1	15	0.233	0.144	0.492	0.189	0.960	0.960
	m2	16	0.189	0.077	0.523	0.174	1.019	0.454
Arctodus simus†	m1	15	2.771	5.723	0.529	0.187	1.042	1.042
	m2	16	5.062	13.835	0.587	0.135	1.029	0.231

†Denotes the extinct taxon; SD, standard deviation, *n*, number of individuals sampled; *Smc*, scale of maximum complexity; $HAsfc_{(3x3)}$, $HAsfc_{(9x9)}$ heterogeneity of complexity in a 3x3 and 9x9 grid, respectively.
