# Supplementary material for: Was the Giant Short-Faced Bear a Hyper-Scavenger? A New Approach to the Dietary Study of Ursids Using Dental Microwear Textures
Source: PLoS One. 2013 Oct 30;8(10):e77531. doi: 10.1371/journal.pone.0077531 (PMC3813673; doi:10.1371/journal.pone.0077531)
Supplement: Table S4 — Table of pairwise differences of Dunn’s procedure for dental microwear attributes of lower first molars of extant ursids. (PDF) [file pone.0077531.s006.pdf]

Table S4. Table of pairwise differences of Dunn's procedure for dental microwear attributes of lower first molars of extant ursids.

	T. ornatus	*U. malayanus*	*U. americanus*	*U. maritimus*
Asfc				
A. melanoleuca	1.83	**27.26***	3.11	-7.30
T. ornatus		**25.43***	1.28	-9.13
U. malayanus			**-24.15***	**-34.56***
U. americanus				-10.41
epLsar				
A. melanoleuca	10.00	-13.00	**12.66***	7.50
T. ornatus		**-23.00***	2.66	-2.50
U. malayanus			**25.66***	**20.50***
U. americanus				-5.16
Smc				
A. melanoleuca	4.63	3.22	-0.91	5.87
T. ornatus		-1.41	-5.54	1.23
U. malayanus			-4.13	2.65
U. americanus				6.78
Tfv				
A. melanoleuca	**-17.20***	**-24.84***	**-21.14***	**-24.67***
T. ornatus		-7.64	-3.94	-7.47
U. malayanus			3.70	0.17
U. americanus				-3.53
HAsfc $_{(3x3)}$				
A. melanoleuca	-13.67	-1.67	**-24.35***	-10.20
T. ornatus		12.00	-10.69	3.47
U. malayanus			**-22.69***	-8.53
U. americanus				**14.15***
HAsfc $_{(9x9)}$				
A. melanoleuca	**-19.07***	0.21	**-18.06***	**-18.13***
T. ornatus		**19.28***	1.01	0.93
U. malayanus			**-18.27***	**-18.34***
U. americanus				-0.08

*Significant values are noted in bold text (P<0.05) and represent analyses performed absent of the Bonferroni correction. *Asfc*, area-scale fractal complexity; *epLsar*, anisotropy; *Smc*, scale of maximum complexity; *Tfv*, textural fill volume; $HAsfc_{(3x3)}$, $HAsfc_{(9x9)}$ heterogeneity of complexity in a 3x3 and 9x9 grid, respectively.
