# Supplementary material for: Was the Giant Short-Faced Bear a Hyper-Scavenger? A New Approach to the Dietary Study of Ursids Using Dental Microwear Textures
Source: PLoS One. 2013 Oct 30;8(10):e77531. doi: 10.1371/journal.pone.0077531 (PMC3813673; doi:10.1371/journal.pone.0077531)
Supplement: Table S5 — Table of pairwise differences (Dunn’s procedure) for lower first molar dental microwear attributes of extant ursids and Arctodus simus. (PDF) [file pone.0077531.s007.pdf]

Table S5. Table of pairwise differences (Dunn's procedure) for lower first molar dental microwear attributes of extant ursids and *Arctodus simus.*

	T. ornatus	*U. malayanus*	*U. americanus*	*U. maritimus*	*Ar. simus*†
Asfc					
A. melanoleuca	2.30	**33.98***	3.80	-9.30	8.30
T. ornatus		**31.68***	1.50	-11.60	6.00
U. malayanus			**-30.17***	**-43.28***	**-25.68***
U. americanus				-13.10	4.50
U. maritimus					**17.60***
epLsar					
A. melanoleuca	14.00	-16.71	**17.72***	10.50	-13.47
T. ornatus		**-30.71***	3.72	-3.50	**-27.47***
U. malayanus			**34.43***	**27.21***	3.25
U. americanus				-7.22	**-31.19***
U. maritimus					**-23.97***
Tfv					
A. melanoleuca	**-19.27***	**-27.43***	**-23.50***	**-27.47***	**-42.67***
T. ornatus		-8.16	-4.23	-8.20	**-23.40***
U. malayanus			3.93	-0.04	-15.24
U. americanus				-3.97	**-19.17***
U. maritimus					-15.20
HAsfc$_{(3x3)}$					
A. melanoleuca	-15.67	-2.07	**-28.00***	-12.47	**-18.87***
T. ornatus		13.60	-12.34	3.20	-3.20
U. malayanus			**-25.94***	-10.40	-16.80
U. americanus				15.54	9.14
U. maritimus					-6.40
HAsfc$_{(9x9)}$					
A. melanoleuca	**-22.13***	0.69	**-21.54***	**-22.20***	**-35.93***
T. ornatus		**22.82***	0.60	-0.07	-13.80
U. malayanus			**-22.22***	**-22.88***	**-36.62***
U. americanus				-0.66	-14.40
U. maritimus					-13.73

*Significant values are noted in bold text (P<0.05) and represent analyses performed absent of the Bonferroni correction. † Denotes the extinct taxon; *Asfc*, area-scale fractal complexity; *epLsar*, anisotropy; *Smc*, scale of maximum complexity; *Tfv*, textural fill volume; *HAsfc*$_{(3x3)}$, *HAsfc*$_{(9x9)}$ heterogeneity of complexity in a 3x3 and 9x9 grid, respectively.
