# Supplementary material for: Was the Giant Short-Faced Bear a Hyper-Scavenger? A New Approach to the Dietary Study of Ursids Using Dental Microwear Textures
Source: PLoS One. 2013 Oct 30;8(10):e77531. doi: 10.1371/journal.pone.0077531 (PMC3813673; doi:10.1371/journal.pone.0077531)
Supplement: Table S6 — Table of pairwise differences (Dunn’s procedure) for extant ursid lower second molar dental microwear attributes, exhibiting no significant differences. (PDF) [file pone.0077531.s008.pdf]

Table S6. Table of pairwise differences (Dunn's procedure) for extant ursid lower second molar dental microwear attributes, exhibiting no significant differences.

	T. ornatus	*U. malayanus*	*U. americanus*	*U. maritimus*
Smc				
A. melanoleuca	2.23	9.61	10.94	7.74
T. ornatus		7.38	8.71	5.51
U. malayanus			1.33	-1.86
U. americanus				-3.20
HAsfc $_{\mathbf{(3x3)}}$				
A. melanoleuca	-3.55	6.85	-8.95	-5.82
T. ornatus		10.39	-5.41	-2.27
U. malayanus			-15.80	-12.67
U. americanus				3.13
HAsfc $_{\mathbf{(9x9)}}$				
A. melanoleuca	3.18	0.65	-1.25	-5.62
T. ornatus		-2.53	-4.43	-8.80
U. malayanus			-1.90	-6.27
U. americanus				-4.37

Smc, scale of maximum complexity; *HAsfc* $_{(3x3)}$, *HAsfc* $_{(9x9)}$ heterogeneity of complexity in a 3x3 and 9x9 grid, respectively.
