# Supplementary material for: Was the Giant Short-Faced Bear a Hyper-Scavenger? A New Approach to the Dietary Study of Ursids Using Dental Microwear Textures
Source: PLoS One. 2013 Oct 30;8(10):e77531. doi: 10.1371/journal.pone.0077531 (PMC3813673; doi:10.1371/journal.pone.0077531)
Supplement: Table S7 — Table of pairwise differences (Dunn’s procedure) for lower second molars of extant ursids and Arctodus simus. (PDF) [file pone.0077531.s009.pdf]

Table S7. Table of pairwise differences (Dunn's procedure) for lower second molars of extant ursids and *Arctodus simus.*

	T. ornatus	*U. malayanus*	*U. americanus*	*U. maritimus*	*Ar. simus*†
Asfc					
A. melanoleuca	-18.18	-15.26	**-35.29***	**-40.47***	**-21.15***
T. ornatus		2.92	**-17.11***	**-22.28***	-2.97
U. malayanus			**-20.03***	**-25.21***	-5.90
U. americanus				-5.18	14.14
U. maritimus					**19.31***
epLsar					
A. melanoleuca	10.73	19.62	**22.59***	**23.27***	**22.64***
T. ornatus		8.89	11.86	12.54	11.92
U. malayanus			2.97	3.65	3.02
U. americanus				0.68	0.05
U. maritimus					-0.63
Tfv					
A. melanoleuca	**-18.55***	-3.97	**-17.04***	-15.45	**-32.32***
T. ornatus		14.58	1.51	3.10	-13.78
U. malayanus			-13.07	-11.48	**-28.35***
U. americanus				1.59	-15.29
U. maritimus					**-16.88***

*Significant values are noted in bold text ($P<0.05$) and represent analyses performed absent of the Bonferroni correction. †Denotes the extinct taxon; *Asfc*, area-scale fractal complexity; *epLsar*, anisotropy; *Tfv*, textural fill volume.
