# Supplementary material for: Was the Giant Short-Faced Bear a Hyper-Scavenger? A New Approach to the Dietary Study of Ursids Using Dental Microwear Textures
Source: PLoS One. 2013 Oct 30;8(10):e77531. doi: 10.1371/journal.pone.0077531 (PMC3813673; doi:10.1371/journal.pone.0077531)
Supplement: Table S8 — Table of pairwise difference for post-hoc tests on significant ( P <0.05) ANOVAs of extant ursid lower first molars. (PDF) [file pone.0077531.s010.pdf]

Table S8. Table of pairwise difference for *post-hoc* tests on significant (*P*<0.05) ANOVAs of extant ursid lower first molars.

	T. ornatus	*U. malayanus*	*U. americanus*	*U. maritimus*
Asfc				
A. melanoleuca	2.00	**27.36****	3.25	-7.2
T. ornatus		**25.36****	1.25	-9.2
U. malayanus			**-24.12****	**-34.56****
U. americanus				-10.45
epLsar				
A. melanoleuca	12.60	-11.80	**16.64***	5.53
T. ornatus		**-24.40****	-4.04	-7.07
U. malayanus			**28.44****	**17.33***
U. americanus				-11.10
Tfv				
A. melanoleuca	**-18.20***	**-25.84****	**-22.14****	**-25.67****
T. ornatus		-7.64	-3.94	-7.47
U. malayanus			3.70	-0.17
U. americanus				-3.53
HAsfc $_{(3x3)}$				
A. melanoleuca	**-13.67***	-1.67	**-24.35****	-10.20
T. ornatus		12.00	-10.69	3.47
U. malayanus			**-22.69***	-8.53
U. americanus				**14.15***
HAsfc $_{(9x9)}$				
A. melanoleuca	**-19.07****	-0.21	**-18.06***	**-18.13***
T. ornatus		**-19.28***	**-1.01***	**-0.93***
U. malayanus			-18.27	-18.34
U. americanus				-0.08

*Significant values (*P*<0.05) based on Fisher's LSD test are noted in bold text**;** **significant values (*P*<0.05) based on both Fisher's LSD and Tukey's HSD tests; *Asfc*, area-scale fractal complexity; *epLsar*, anisotropy; *Smc*, scale of maximum complexity; *Tfv*, textural fill volume; *HAsfc* $_{(3x3)}$, *HAsfc* $_{(9x9)}$ heterogeneity of complexity in a 3x3 and 9x9 grid, respectively.
