# Supplementary material for: Was the Giant Short-Faced Bear a Hyper-Scavenger? A New Approach to the Dietary Study of Ursids Using Dental Microwear Textures
Source: PLoS One. 2013 Oct 30;8(10):e77531. doi: 10.1371/journal.pone.0077531 (PMC3813673; doi:10.1371/journal.pone.0077531)
Supplement: Table S9 — Table of pairwise difference for post-hoc tests on significant ( P <0.05) ANOVAs of extant ursid lower second molars. (PDF) [file pone.0077531.s011.pdf]

Table S9. Table of pairwise difference for *post-hoc* tests on significant (*P*<0.05) ANOVAs of extant ursid lower second molars.

	T. ornatus	*U. malayanus*	*U. americanus*	*U. maritimus*
Asfc				
A. melanoleuca	**-13.00***	-11.20	**-26.36****	**-29.86****
T. ornatus		1.80	**-13.36***	**-16.86****
U. malayanus			**-15.17***	**-18.67****
U. americanus				-3.50
epLsar				
A. melanoleuca	8.91	15.99	**17.75***	**18.51****
T. ornatus		7.08	8.84	9.60
U. malayanus			1.77	2.52
U. americanus				0.75

*Significant values (*P*<0.05) based on Fisher's LSD test**;** **significant values (*P*<0.05) based on both Fisher's LSD and Tukey's HSD tests; *Asfc*, area-scale fractal complexity; *epLsar*, anisotropy.
