# Supplementary material for: Was the Giant Short-Faced Bear a Hyper-Scavenger? A New Approach to the Dietary Study of Ursids Using Dental Microwear Textures
Source: PLoS One. 2013 Oct 30;8(10):e77531. doi: 10.1371/journal.pone.0077531 (PMC3813673; doi:10.1371/journal.pone.0077531)
Supplement: Table S10 — Data for lower first (m1) and second (m2) lower molars of analyzed specimens. (PDF) [file pone.0077531.s012.pdf]

Table S10. Data for lower first (m1) and second (m2) lower molars of analyzed specimens.

Taxon	Specimen Number	Tooth	Location	*Asfc*	*epLsar*	*Smc*	*Tfv*	*Hasfc* (3x3)	*Hasfc* (9x9)
Ailuropoda melanoleuca	NMNH 258423	m1	China	1.987	0.00348	0.2670	27.955	0.4462	0.6647
Ailuropoda melanoleuca	NMNH 258425	m1	China	1.422	0.00491	0.1539	0.000	0.3693	0.6956
Ailuropoda melanoleuca	NMNH 258834	m1	China	2.701	0.00699	0.2712	0.000	0.5976	0.8401
Ailuropoda melanoleuca	NMNH 258835	m1	China	2.871	0.00715	0.2669	168.21	0.3898	0.5831
Ailuropoda melanoleuca	NMNH 258836	m1	China	6.592	0.00644	0.2668	10971	0.2551	0.7078
Ailuropoda melanoleuca	NMNH 259027	m1	China	2.351	0.00154	0.2667	12551	0.4117	0.4676
Ailuropoda melanoleuca	NMNH 259028	m1	China	2.617	0.00380	0.2725	0.000	0.3948	0.6649
Ailuropoda melanoleuca	NMNH 259029	m1	China	6.593	0.00481	0.1511	0.000	0.2474	0.3040
Ailuropoda melanoleuca	NMNH 259074	m1	China	14.070	0.00363	0.1521	15034	0.3835	0.6610
Ailuropoda melanoleuca	NMNH 259075	m1	China	2.836	0.00602	0.5081	0.000	0.5229	0.6328
Ailuropoda melanoleuca	NMNH 259076	m1	China	1.973	0.00556	0.2674	111.99	0.4227	0.8316
Ailuropoda melanoleuca	NMNH 259400	m1	China	5.511	0.00277	0.1505	1588.7	0.3730	0.5581
Ailuropoda melanoleuca	NMNH 259401	m1	China	2.645	0.00655	0.4165	0.000	0.4494	0.7484
Ailuropoda melanoleuca	NMNH 259402	m1	China	22.346	0.00242	0.1499	9017.5	0.1742	0.2605
Ailuropoda melanoleuca	NMNH 399447	m1	China	9.065	0.00426	0.1499	3324.9	0.3599	0.4823
Tremarctos ornatus	AMNH 149032	m1	Columbia	13.066	0.00227	0.1530	15148	0.6390	0.8731
Tremarctos ornatus	AMNH 67732	m1	Ecuador	7.331	0.00311	0.1526	10468	0.3929	0.8166
Tremarctos ornatus	AMNH 99308	m1	Peru	3.664	0.00227	0.1560	14913	0.3661	0.6952
Tremarctos ornatus	NMNH 155575	m1	Venezuela	3.931	0.00207	0.2724	269.60	0.5591	0.6218
Tremarctos ornatus	NMNH 168115	m1	Venezuela	2.337	0.00400	0.3500	12486	0.4869	0.7232
Tremarctos ornatus	NMNH 170656	m1	Venezuela	1.420	0.00418	0.3415	6959.0	0.4493	0.6693
Tremarctos ornatus	NMNH 170657	m1	Venezuela	1.812	0.00161	0.1499	6056.4	0.4453	0.6812
Tremarctos ornatus	NMNH 171011	m1	Venezuela	6.119	0.00237	0.1560	14151	0.4991	0.8622
Tremarctos ornatus	NMNH 194309	m1	Peru	2.171	0.00472	0.1562	6636.2	0.4120	0.9376
Tremarctos ornatus	NMNH 210321	m1	Ecuador	8.691	0.00181	0.1515	13340	0.7332	1.2423
Tremarctos ornatus	NMNH 210322	m1	Ecuador	3.662	0.00328	0.1529	7177.1	0.3618	0.8051
Tremarctos ornatus	NMNH 210323	m1	Ecuador	4.387	0.00392	0.1517	28.262	0.3134	0.6721

Taxon	Specimen Number	Tooth	Location	*Asfc*	*epLsar*	*Smc*	*Tfv*	*Hasfc* (3x3)	*Hasfc* (9x9)
Tremarctos ornatus	NMNH 210324	m1	Ecuador	1.140	0.00533	0.2702	2375.9	0.4816	0.6842
Tremarctos ornatus	NMNH 271418	m1	Bolivia	2.497	0.00410	0.2122	4954.2	0.6162	1.0666
Tremarctos ornatus	NMNH 582002	m1	Unknown	2.985	0.00555	0.3414	13190	0.4232	0.7978
Ursus americanus	NMNH 176594	m1	Alaska	6.587	0.00270	0.1538	16086	0.5402	0.8831
Ursus americanus	NMNH 177657	m1	Alaska	1.588	0.00504	0.3443	10060	0.4827	0.7672
Ursus americanus	NMNH 177659	m1	Alaska	5.215	0.00416	0.1502	14947	0.6051	0.7135
Ursus americanus	NMNH 180277	m1	Alaska	2.152	0.00187	0.2094	10320	0.7909	1.0287
Ursus americanus	NMNH 231506	m1	Alaska	8.985	0.00180	0.1499	12016	0.4903	1.0647
Ursus americanus	NMNH 231507	m1	Alaska	5.714	0.00196	0.1509	7815.4	0.4753	0.8225
Ursus americanus	NMNH 231509	m1	Alaska	2.994	0.00377	0.2083	2867.1	0.4484	0.7275
Ursus americanus	NMNH 231510	m1	Alaska	3.784	0.00402	0.2678	10524	0.4164	0.6837
Ursus americanus	UF 13875	m1	Florida	2.121	0.00310	0.2670	7165.9	0.3665	0.4887
Ursus americanus	UF 13876	m1	Florida	11.138	0.00283	0.1602	16790	0.7342	1.2994
Ursus americanus	UF 28416	m1	Florida	1.836	0.00320	0.1586	5927.9	0.5646	0.6305
Ursus americanus	UF 28423	m1	Florida	2.665	0.00213	0.2697	10692	0.4234	0.6519
Ursus americanus	UF 28436	m1	Florida	2.818	0.00361	0.3414	9963.4	0.6456	0.7932
Ursus americanus	UF 28445	m1	Florida	1.551	0.00308	0.4194	7850.4	0.4605	0.5162
Ursus americanus	UF 28449	m1	Florida	7.567	0.00145	0.1602	14182	0.6222	2.0846
Ursus americanus	UF 6496	m1	Florida	0.603	0.00172	0.4209	2665.7	0.7638	0.8480
Ursus malayanus	NMNH 123138	m1	Unknown	0.556	0.00217	0.4164	8788.6	0.3784	0.5129
Ursus malayanus	NMNH 151866	m1	Unknown	1.703	0.00147	0.1499	7236.7	0.3733	0.6833
Ursus malayanus	NMNH 197254	m1	Malay Peninsula	1.125	0.00348	0.5090	8105.7	0.4658	0.5149
Ursus malayanus	NMNH 198713	m1	Malay Peninsula	2.753	0.00235	0.1499	12992	0.3349	0.6473
Ursus malayanus	NMNH 198714	m1	Malay Peninsula	1.563	0.00338	0.2665	11540	0.4433	0.7195
Ursus malayanus	NMNH 198715	m1	Malay Peninsula	1.563	0.00500	0.1500	16750	0.3938	0.5469
Ursus malayanus	NMNH 358645	m1	Malay Peninsula	0.807	0.00351	0.2665	11989	0.4309	0.7699
Ursus maritimus	NMNH 212589	m1	Alaska	8.124	0.00148	0.1499	7247.4	0.6064	1.2627
Ursus maritimus	NMNH 212590	m1	Alaska	4.914	0.00275	0.2671	11467	0.8556	1.1787
Ursus maritimus	NMNH 215714	m1	Alaska	5.521	0.00469	0.2716	11864	0.3699	0.5861

Taxon	Specimen Number	Tooth	Location	*Asfc*	*epLsar*	*Smc*	*Tfv*	*Hasfc* (3x3)	*Hasfc* (9x9)
Ursus maritimus	NMNH 227099	m1	Alaska	3.156	0.00254	0.2097	13633	0.4422	0.7135
Ursus maritimus	NMNH2 27105	m1	Alaska	3.285	0.00120	0.2675	14374	0.3764	0.5318
Ursus maritimus	NMNH 265099	m1	Alaska	5.937	0.00557	0.1499	12088	0.3804	0.5312
Ursus maritimus	NMNH 512111	m1	N. Hudson Bay	4.159	0.00591	0.1083	10200	0.5200	1.2690
Ursus maritimus	NMNH 512113	m1	N. Hudson Bay	6.794	0.00222	0.1500	4734.8	0.5824	1.2929
Ursus maritimus	NMNH 512117	m1	N. Hudson Bay	2.464	0.00457	0.2687	9294.7	0.3400	0.7726
Ursus maritimus	NMNH 512121	m1	N. Hudson Bay	4.814	0.00297	0.2123	10230	0.5483	1.4642
Ursus maritimus	NMNH 512130	m1	N. Hudson Bay	2.959	0.00671	0.1516	11618	0.3285	0.6682
Ursus maritimus	NMNH 512133	m1	N. Hudson Bay	2.944	0.00455	0.7085	15200	0.3322	0.6304
Ursus maritimus	NMNH 512136	m1	N. Hudson Bay	11.556	0.00384	0.1516	14350	0.4721	0.9040
Ursus maritimus	NMNH 512151	m1	N. Hudson Bay	3.086	0.00376	0.1517	1176.6	0.9245	2.0794
Ursus maritimus	NMNH 512163	m1	N. Hudson Bay	4.003	0.00306	0.2670	12269	0.3007	0.5191
Arctodus simus†	LACMHC 1292	m1	Pit 77	2.738	0.00222	0.1499	13393	0.3037	0.7873
Arctodus simus†	LACMHC 401	m1	Pit 77	7.340	0.00248	0.1512	16444	0.5174	0.8740
Arctodus simus†	LACMHC 57520	m1	Pit unknown	2.261	0.00259	0.2108	7248.6	0.3261	0.8002
Arctodus simus†	LACMHC 57521	m1	Pit unknown	1.730	0.00148	8.9601	13345	0.3310	0.6494
Arctodus simus†	LACMHC 618	m1	Pit 9	0.778	0.00342	19.131	12196	0.4448	0.8489
Arctodus simus†	LACMHC 619	m1	Pit 9	4.747	0.00202	0.1499	16327	0.7396	1.5934
Arctodus simus†	LACMHC 626	m1	Pit 9	2.117	0.00307	0.4330	13333	0.3543	0.6771
Arctodus simus†	LACMHC 86	m1	Pit 9	4.009	0.00156	0.1529	12484	0.8432	1.4995
Arctodus simus†	LACMHC 88	m1	Pit 9	2.933	0.00403	0.1503	10557	0.6541	1.2913
Arctodus simus†	LACMHC 90	m1	Pit 9	1.148	0.00229	11.270	13221	0.2911	0.7892
Arctodus simus†	LACMHC 52234	m1	Pit 91	3.732	0.00232	0.1506	13983	0.5878	1.4039
Arctodus simus†	LACMHC Z-19	m1	Pit unknown	2.590	0.00215	0.1509	15612	0.6007	1.0087
Arctodus simus†	LACMHC Z-7	m1	Pit unknown	4.913	0.00203	0.1501	14304	0.8315	1.5095
Arctodus simus†	LACMHC Z-8	m1	Pit unknown	2.166	0.00376	0.2086	11384	0.5811	1.0590
Arctodus simus†	LACMHC Z-9	m1	Pit unknown	7.048	0.00168	0.1502	16893	0.5331	0.8321
Ailuropoda melanoleuca	NMNH 258423	m2	China	1.915	0.00498	0.2669	14591	0.5642	1.0737
Ailuropoda melanoleuca	NMNH 258425	m2	China	1.047	0.00330	0.1508	1782.9	0.5301	0.9328

Taxon	Specimen Number	Tooth	Location	*Asfc*	*epLsar*	*Smc*	*Tfv*	*Hasfc* (3x3)	*Hasfc* (9x9)
Ailuropoda melanoleuca	NMNH 258834	m2	China	3.895	0.00037	0.2082	12470	0.3092	0.7228
Ailuropoda melanoleuca	NMNH 258836	m2	China	1.920	0.00366	0.1524	12142	0.4339	0.7150
Ailuropoda melanoleuca	NMNH 259027	m2	China	2.530	0.00295	0.1547	4435.8	0.4472	1.0987
Ailuropoda melanoleuca	NMNH 259028	m2	China	1.680	0.00828	0.2093	210.98	0.4260	0.6474
Ailuropoda melanoleuca	NMNH 259074	m2	China	1.849	0.00434	0.1535	11433	0.5789	1.1577
Ailuropoda melanoleuca	NMNH 259076	m2	China	2.794	0.00301	0.1509	11593	0.3783	0.5550
Ailuropoda melanoleuca	NMNH 259401	m2	China	3.015	0.00215	0.1516	1790.6	0.3273	0.6390
Ailuropoda melanoleuca	NMNH 259402	m2	China	0.693	0.00650	0.4166	14492	0.6084	0.8103
Ailuropoda melanoleuca	NMNH3 99447	m2	China	0.618	0.00355	0.2699	5585.8	0.3847	0.5733
Tremarctos ornatus	AMNH 149302	m2	Ecuador	1.256	0.00259	0.2691	19630	0.4969	0.5849
Tremarctos ornatus	AMNH 67732	m2	Peru	4.478	0.00239	0.1504	9841.2	0.5786	0.9427
Tremarctos ornatus	AMNH 99308	m2	Venezuela	7.596	0.00427	0.1513	16786	0.8624	1.1349
Tremarctos ornatus	NMNH 155575	m2	Venezuela	2.549	0.00401	0.4164	15199	0.4402	0.4766
Tremarctos ornatus	NMNH 170656	m2	Venezuela	3.016	0.00335	0.1503	11925	0.4916	0.8569
Tremarctos ornatus	NMNH 171011	m2	Ecuador	3.538	0.00229	0.1513	4029.1	0.3663	0.5863
Tremarctos ornatus	NMNH 210321	m2	Ecuador	3.190	0.00306	0.4177	15475	0.3794	0.5950
Tremarctos ornatus	NMNH 210323	m2	Ecuador	2.068	0.00144	0.2670	9451.1	0.4808	0.7074
Tremarctos ornatus	NMNH 210324	m2	Bolivia	3.683	0.00317	0.1549	16907	0.5771	0.7017
Tremarctos ornatus	NMNH 271418	m2	Unknown	6.037	0.00187	0.2093	10601	0.3832	0.7301
Tremarctos ornatus	NMNH 582002	m2	Alaska	8.478	0.00185	0.1502	13012	0.3353	0.9791
Ursus americanus	NMNH 177657	m2	Alaska	13.130	0.00420	0.1500	16868	0.4618	0.9099
Ursus americanus	NMNH 177659	m2	Alaska	10.621	0.00250	0.1499	15787	0.4356	0.6009
Ursus americanus	NMNH 180277	m2	Alaska	7.140	0.00082	0.1507	3140.3	0.2900	0.3481
Ursus americanus	NMNH 231506	m2	Alaska	10.717	0.00207	0.1512	16432	0.3826	0.5825
Ursus americanus	NMNH 231507	m2	Alaska	6.659	0.00251	0.1514	13262	0.4810	0.9153
Ursus americanus	NMNH 231509	m2	Alaska	3.788	0.00454	0.2101	4561.0	0.4697	0.5776
Ursus americanus	NMNH 231510	m2	Florida	16.550	0.00140	0.1510	16630	0.4486	0.6169
Ursus americanus	UF 13875	m2	Florida	6.792	0.00188	0.1502	16981	0.7130	1.4092
Ursus americanus	UF 13876	m2	Florida	6.357	0.00326	0.1512	14230	0.5661	0.9024

Taxon	Specimen Number	Tooth	Location	*Asfc*	*epLsar*	*Smc*	*Tfv*	*Hasfc* (3x3)	*Hasfc* (9x9)
Ursus americanus	UF 28416	m2	Florida	1.874	0.00080	0.3448	6910.1	0.5206	0.6524
Ursus americanus	UF 28423	m2	Florida	14.572	0.00212	0.1514	14416	0.6282	0.7762
Ursus americanus	UF 28436	m2	Florida	9.712	0.00133	0.1553	12894	0.4030	0.8318
Ursus americanus	UF 28445	m2	Florida	4.189	0.00097	0.1517	9240.2	0.8290	1.0481
Ursus americanus	UF 28449	m2	Florida	4.084	0.00192	0.1533	10773	0.6844	1.0903
Ursus americanus	UF 6496	m2	Unknown	1.530	0.00273	0.2083	10748	0.7309	1.7815
Ursus malayanus	NMNH 123138	m2	Unknown	1.052	0.00204	0.2668	4242.6	0.2846	0.4738
Ursus malayanus	NMNH 151866	m2	Malay Peninsula	3.280	0.00123	0.1502	13214	0.6156	0.9778
Ursus malayanus	NMNH 197254	m2	Malay Peninsula	1.325	0.00379	0.3428	8850.8	0.3783	0.6765
Ursus malayanus	NMNH 198713	m2	Malay Peninsula	6.703	0.00267	0.1502	10240	0.2851	0.6790
Ursus malayanus	NMNH 198715	m2	Malay Peninsula	6.762	0.00214	0.1504	13791	0.2893	0.8016
Ursus malayanus	NMNH 358645	m2	Alaska	4.664	0.00189	0.1517	13494	0.6328	1.1585
Ursus maritimus	NMNH 212589	m2	Alaska	8.971	0.00116	0.1509	11306	0.8363	1.0155
Ursus maritimus	NMNH 212590	m2	Alaska	4.802	0.00102	0.1503	11528	0.3884	0.5716
Ursus maritimus	NMNH 215714	m2	Alaska	5.645	0.00120	0.1501	13844	0.4808	0.6554
Ursus maritimus	NMNH 227099	m2	Alaska	9.140	0.00371	0.2096	13518	0.7349	1.6357
Ursus maritimus	NMNH 227105	m2	Alaska	11.314	0.00127	0.1500	10574	0.6670	1.5761
Ursus maritimus	NMNH 265099	m2	Alaska	12.422	0.00075	0.1536	15123	0.5081	0.7088
Ursus maritimus	NMNH 336269	m2	N. Hudson Bay	6.474	0.00150	0.1518	10850	0.3782	0.8140
Ursus maritimus	NMNH 512111	m2	N. Hudson Bay	2.381	0.00580	0.2699	10663	0.5042	0.5617
Ursus maritimus	NMNH 512113	m2	N. Hudson Bay	9.243	0.00230	0.1512	11065	0.4533	1.0375
Ursus maritimus	NMNH 512117	m2	N. Hudson Bay	1.740	0.00338	0.4346	13472	0.4831	0.9181
Ursus maritimus	NMNH 512121	m2	N. Hudson Bay	11.210	0.00250	0.1631	12454	0.7838	1.9848
Ursus maritimus	NMNH 512130	m2	N. Hudson Bay	6.813	0.00137	0.1502	13831	0.3735	0.8954
Ursus maritimus	NMNH 512133	m2	N. Hudson Bay	14.690	0.00297	0.1586	14707	0.3353	0.8858
Ursus maritimus	NMNH 512136	m2	N. Hudson Bay	12.418	0.00239	0.1499	18800	0.3349	0.5751
Ursus maritimus	NMNH 512151	m2	N. Hudson Bay	8.960	0.00124	0.1557	13244	0.7508	1.7131
Ursus maritimus	NMNH 512163	m2	N. Hudson Bay	9.564	0.00279	0.2723	13599	0.3526	0.7551

Taxon	Specimen Number	Tooth	Location	*Asfc*	*epLsar*	*Smc*	*Tfv*	*Hasfc* (3x3)	*Hasfc* (9x9)
Arctodus simus†	LACMHC 1292	m2	Pit 77	3.902	0.00102	0.2083	12311	0.6179	0.9883
Arctodus simus†	LACMHC 401	m2	Pit 77	6.117	0.00118	0.2684	17473	0.6191	0.9840
Arctodus simus†	LACMHC 57514	m2	Pit unknown	2.799	0.00414	0.1500	14683	0.3650	0.6985
Arctodus simus†	LACMHC 57520	m2	Pit unknown	8.292	0.00195	0.1511	14847	0.5684	1.0872
Arctodus simus†	LACMHC 57521	m2	Pit 9	2.073	0.00100	0.1518	16432	0.6709	1.4129
Arctodus simus†	LACMHC 618	m2	Pit 9	2.364	0.00220	0.2086	14033	0.8065	1.5074
Arctodus simus†	LACMHC 619	m2	Pit 9	2.960	0.00278	0.1507	15756	0.6958	1.0301
Arctodus simus†	LACMHC 626	m2	Pit 9	5.151	0.00232	0.1505	15409	0.4781	1.2626
Arctodus simus†	LACMHC 86	m2	Pit 9	4.327	0.00165	49.531	15380	0.4558	0.8271
Arctodus simus†	LACMHC 88	m2	Pit 9	7.483	0.00286	0.1513	16416	0.4638	1.0642
Arctodus simus†	LACMHC 89	m2	Pit 9	3.532	0.00198	0.1503	12419	0.5638	0.9382
Arctodus simus†	LACMHC 90	m2	Pit 91	1.160	0.00362	28.760	11985	0.7688	0.9913
Arctodus simus†	LACMHC 52234	m2	Pit unknown	2.153	0.00195	0.5083	15868	0.3804	0.6366
Arctodus simus†	LACMHC Z-19	m2	Pit unknown	7.093	0.00377	0.1533	16156	0.5934	0.9008
Arctodus simus†	LACMHC Z-7	m2	Pit unknown	6.952	0.00143	0.1510	17652	0.7714	0.9502
Arctodus simus†	LACMHC Z-9	m2	Pit unknown	7.026	0.00090	0.1522	13622	0.5660	1.1850

AMNH, American Museum of Natural History, New York, NY; NMNH, Smithsonian Institute National Museum of Natural History, Washington D.C.; FLMNH, Florida Museum of Natural History, Gainesville, FL; LACMHC, Los Angeles County Museum of Natural History, Page Museum, Hancock Collection, Los Angeles, CA; † denotes the extinct taxon; *Asfc*, area-scale fractal complexity; *epLsar*, anisotropy; *Smc*, scale of maximum complexity; *Tfv*, textural fill volume; $HAsfc_{(3x3)}$, $HAsfc_{(9x9)}$ heterogeneity of complexity in a 3x3 and 9x9 grid, respectively.
