# Supplementary material for: Was the Giant Short-Faced Bear a Hyper-Scavenger? A New Approach to the Dietary Study of Ursids Using Dental Microwear Textures
Source: PLoS One. 2013 Oct 30;8(10):e77531. doi: 10.1371/journal.pone.0077531 (PMC3813673; doi:10.1371/journal.pone.0077531)
Supplement: File S1 — Supplemental dietary background of extant Ursids. (PDF) [file pone.0077531.s013.pdf]

Supplemental Dietary Background of Extant Ursids

Ursus maritimus

The carnivorous polar bear, *Ursus maritimus* is considered a hyper-carnivore that actively preys on marine mammals in Arctic waters. The primary food source of polar bears is the ringed seal (*Phoca hispida*), and bears mainly focus on the blubber and flesh of their prey [1]. Polar bears also prey upon harbor seals (*P. vitalina*), the larger bearded seals (*Erignathus barbatus*), walruses (*Odobenus rosmarus*), and occasionally narwhal (*Monodon monoceras*) [1], [2]. Larger male polar bears are better suited for hunting bigger prey items than smaller females, who instead focus on smaller bodied seal species, and juveniles.

Polar bears engage in some scavenging of whale carcasses and consume terrestrial food sources to supplement marine prey resources. Scavenging is becoming more frequent with earlier seasonal break up of Arctic sea ice, which polar bears rely on for hunting [2]. Typically, polar bears fast while they are on land during the summer months, and historic observations indicated that terrestrial food sources were negligible in the diet [1]. However, rare reports exist of bears hunting large bodied terrestrial herbivores [1], and Arctic charr (*Salvelinus alpinus,* a fresh water fish in the salmon family) is sometimes consumed [3]. Bears found over ten kilometers inland from northwest Hudson Bay occasionally consume berries, moss, and grasses during the summer months [4]. It is hypothesized that as sea ice breaks up earlier, and *U. maritimus* cannot store adequate fat reserves through seal hunting, terrestrial food sources will become increasingly important in their diet [3].

Ursus americanus

The American black bear, *Ursus americanus* is an opportunistic omnivore. Its diet is highly variable across seasons and geographic space. Broadly, spring diets include sprouting young vegetation, carrion (animal carcasses), and insects. In summer, fruits and herbaceous plants are important (e.g. dandelion), and in autumn, berries (soft mast) and nuts (hard mast) are the primary food items [5]. Animal matter, including carrion, small vertebrates, and insects, are consumed opportunistically throughout the year. Black bears also hunt larger vertebrates (e.g. white-tailed deer, *Odocoileus virginianus*) on rare occasions [5]. In Florida, saw palmetto hearts (*Serenoa repens*) are a significant food source for *U. americanus* year round, and insects are also important, particularly in the fall when beetles and yellow-jackets are consumed in high abundance [6]. Because black bears are opportunistic omnivores, their diet is highly adaptable in years of changing food availabilities [5], [7], which may allow them to survive food-stressed periods.

Ursus malayanus

The insectivorous/frugivorous/omnivorous Malayan sun bear, *Ursus malayanus*, is found in the dense forests of Malaysia. Overall, *U. malayanus* relies mainly on insects (termites, ants, beetle larvae, cockroaches, bees), which are some of the most stable food sources in tropical forests [8]. Other important food sources include figs (*Ficus* sp., present year round), flowers, honey, leafy vegetation and small vertebrates [8], [9].

The vegetation of the Malayan region is strongly influenced by El Niño Southern Oscillations, which prompt mass synchronous fruiting events through a mechanism that is not well understood, but occurs every 2–15 years [9]. During mass fruiting, more than 15% of tree species produce an overabundance of fruit for a short time period, followed by a fruit scarcity lasting months to years [9]. The sun bear responds to mass fruiting events by consuming a diet of up to 100% fruit, typically of tree fruits that are fleshy and fibrous. Bears avoid eating large internal fruit pits and consume only those fruits with the highest nutritional value, while avoiding those of lower nutritional content that are found year-round (e.g., figs) [9]. This behavior allows *U. malayanus* to effectively store fat, which aids in survival during long periods of fruit scarcity. The behavioral switch to becoming an insectivorous/omnivorous bear allows for survival in a habitat where food sources are both less nutritious and less available. The evolution of these behavioral characteristics was likely a critical factor that enabled *U. malayanus* to persist through time, demonstrating the importance of dietary adaptability in the evolutionary history of this species.

Tremarctos ornatus

The spectacled bear, *Tremarctos ornatus*, is an herbivorous/omnivorous bear found in the Andes Mountains of South America. It is a capable tree climber, and prefers dense forest cover [10]. Overall, about 75% of its diet is composed of plant matter [10]. The largest plant contributors are bromeliads (family: Bromeliaceae, up to 22 species consumed). During the non-fruiting season, *T.* ornatus almost exclusively consumes tough, succulent bromeliad hearts, which are eaten following removal of the hard plant exteriors [10]. Bromeliads are also important because they contain up to one liter of water, which may be particularly crucial to bears inhabiting coastal desert scrub and steppe habitats [10]. During the fruiting season, figs (*Ficus* sp.) and capers (*Capparis* sp.) are significant food sources, along with numerous other succulent tree fruits. Additional vegetation food sources include cactus, palm frond petioles, shrub berries, and tree wood [10]. Spectacled bears will also consume insects and honey in addition to bromeliad hearts during the non-fruiting season [10]. Despite *T. ornatus* feeding primarily on plant resources, it will occasionally feed on animal matter including small vertebrates (e.g. rodents, lizards) and infrequently, larger vertebrates (e.g. goats, cattle, turtles), though larger vertebrates are usually encountered as carrion.

Ailuropoda melanoleuca

The giant panda, *Ailuropoda melanoleuca*, is a specialist bamboo consumer. Giant pandas inhabit the Qinling Mountains and Sichuan area of China and will seasonally migrate up and down elevation with the availability of several bamboo species [11]. *Ailuropoda melanoleuca* individuals have unique features associated with bamboo consumption, including broad, flat, cuspidate premolars and molars, and an enlarged wrist bone (radial sesamoid) which functions similar to an opposable thumb to allow grasping of bamboo [12].

Giant pandas are highly selective foragers and utilize different parts of bamboo across different seasons. Leaves are present year round, and are the primary food source of *A. melanoleuca* for the majority of the year [13]. A panda will strip leaves from

a branch by pulling the entire branch through its anterior teeth, and forming a wad of leaves at the corner of its mouth. This wad is then held in the paw and bites are taken as the wad is consumed as a whole [11], [13]. In the spring, *A. melanoleuca* switches to consumption of pith (interior layers of the culm, or the major stem), likely due to the higher lignin content of leaves at this time [11], or perhaps a change in the chemical composition of the leaves [13]. Consumption of pith occurs following peeling of the hard culm exterior. An individual holds the bamboo stem with its paw and uses its anterior molars to crack and then strip off the hard outer casing. Following peeling, the pith is bitten off and chewed [11], [13]. During the season of pith consumption, newly emerged soft shoots are also eaten, likely to supplement pith, which is less nutritious than leaves [13]. Overall, *A. melanoleuca* has the most specialized diet of all extant bears.

References

1. Thiemann GW, Iverson SJ, Stirling I (2008) Polar bear diets and arctic marine food webs: Insights from fatty acid analysis. J Mammal 78: 591–613.
2. Bentzen TW, Follmann EH, Amstrup SC, York GS, Wooller MJ, et al. (2007) Variation in winter diet of southern Beaufort Sea polar bears inferred from stable isotope analysis. Can J Zool 85: 596–608.
3. Dyck, MG, Kebreab E (2009) Estimating the energetic contribution of polar bear (*Ursus maritimus*) summer diets to the total energy budget. J Mammal 90: 585–593.
4. Derocher AE, Andriashek D, Stirling I (1993) Terrestrial foraging by polar bears during the ice-free period in western Hudson Bay. Arctic 46: 251–254.
5. Raine RM, Kansas JL (1990) Black bear seasonal food habits and distribution by elevation in Banff National Park, Alberta. Bears: Their biology and management 8: 297–304.
6. Stratman MR, Pelton MR (1999) Feeding ecology of black bears in northwest Florida. Florida Field Naturalist 27(3): 95–102.
7. Roof JC (1997) Black bear food habits in the Lower Wekiva River basin of central Florida. Florida Field Nat 25: 92–97.
8. Wong S, Servheen C, Ambu L (2002) Food habits of malayan sun bears in lowland tropical forests of Borneo. Ursus 13: 127–136.
9. Fredriksson GM, Wich SA, Trisno (2006) Frugivory in sun bears (*Helarctos malayanus*) is linked to El Niño-related fluctuations in fruiting phenology, East Kalimantan Indonesia. Biol J Linnean Soc 89: 489–508..
10. Peyton B (1980) Ecology, distribution, and food habits of spectacled bears, *Tremarctos ornatus*, in Peru. J Mammal 61: 639-652.
11. Long Y, Lu Z, Wang D, Zhu X, Wang H, et al. (2004) Nutritional strategy of giant pandas in the Qinling Mountains of China. In: Lindburg D, Baragona K, editors. Giant Pandas: Biology and Conservation. University of California Press. pp. 103-116.

12. Schaller GB, Hu J, Pan W, Zhu J (1985) The Giant Pandas of Wolong. Chicago, Illinois: University of Chicago Press. 298p.

13. Hansen RL, Carr MM, Apanavicius CJ, Jiang P, Bissell HA, et al. (2010) Seasonal shifts in giant panda feeding behavior: Relationships to bamboo plant part consumption. Zoo Biol 29: 470–83.
